# Beyond Weight Loss: Glucagon-Like Peptide-1 Receptor Agonists and Antipsychotic Adherence in Schizophrenia

**DOI:** 10.7759/cureus.114933

**Published:** 2026-08-21

**Authors:** Iván López-Ibor, Hugh Humphery, Gustavo Tafet

**Affiliations:** 1 Department of Psychiatry, South Florida Integrative Medicine, Miami, USA; 2 Department of Psychiatry, López Ibor Clinic, Madrid, ESP; 3 Department of Psychology, Dr. Esquerdo Sanatorium, Madrid, ESP; 4 Department of Psychiatry, Dr. Leon's Clinic, Madrid, ESP; 5 Department of Psychiatry, Herbert Wertheim College of Medicine, Florida International University, Miami, USA; 6 Department of Psychiatry, Texas A&amp;M University - Central Texas, Killeen, USA

**Keywords:** antipsychotic-induced weight gain, cardiometabolic risk, glucagon-like peptide-1 receptor agonists, medication adherence, schizophrenia, semaglutide

## Abstract

People with schizophrenia experience markedly reduced life expectancy, driven largely by cardiometabolic disease, and several of the most effective antipsychotics are also among those most likely to promote weight gain. Weight gain is among the adverse effects patients find least acceptable and one they frequently cite when they abandon treatment, which links metabolic harm directly to the non-adherence that precipitates relapse. Glucagon-like peptide-1 receptor agonists, now supported by randomized trials in schizophrenia-spectrum populations, reduce weight and metabolic burden without destabilizing psychiatric symptoms. In this editorial, we argue that their most consequential effect in serious mental illness may not be the weight they remove but the treatment they help patients keep taking: by neutralizing the adverse effect patients most resent, these agents may protect antipsychotic adherence itself. We call for trials that adopt adherence and discontinuation as endpoints and for psychiatry to reclaim ownership of cardiometabolic prescribing.

## Editorial

Schizophrenia, a chronic psychotic disorder, carries a burden that extends well beyond its psychiatric symptoms. The persistence of a 15-year mortality gap in schizophrenia is one of the quieter scandals of contemporary medicine [1]. That gap has proved stubborn, and in several health systems it has widened rather than closed [1]. Contrary to a common assumption, the excess deaths are driven not primarily by suicide but predominantly by cardiovascular disease, type 2 diabetes, and the metabolic derangements that precede them [1,2] - a burden of preventable medical comorbidity, captured in comparative data on the differential metabolic toxicity of individual antipsychotics [2], that is best understood broadly rather than as a single cardiometabolic number. The uncomfortable implication is that psychiatry has grown steadily better at controlling psychotic symptoms [1] while failing at the more elementary task of keeping the patient alive [1].

Part of the explanation lies in our own prescriptions. The agents that work best for the most severe illness - clozapine above all, and olanzapine close behind, both second-generation (atypical) antipsychotics whose comparative metabolic liability has been systematically documented [2] - are precisely those that impose the greatest weight gain and the steepest deterioration in glucose and lipids [2-4]. This is the therapeutic paradox of modern psychopharmacology: efficacy and metabolic toxicity travel together, and the patients who most need the effective drug are the ones most exposed to its harm. We have responded, reasonably, with metformin - a biguanide that improves insulin sensitivity - with lifestyle programs, and with closer metabolic monitoring [5]. The results have been real but modest, a limitation that has helped motivate interest in agents with a different mechanism of action [5].

There is, however, a second cost that is less often counted, because it does not appear on a metabolic panel - a cost this editorial addresses directly [5]. For many patients, weight gain is not an abstract cardiovascular risk factor to be managed over decades; it is the immediate, visible, socially punishing consequence of taking the medication, and it is among the reasons they most often give for stopping, contributing to the treatment discontinuation that this editorial links directly to metabolic burden [5]. Here the metabolic and the psychiatric collapse into a single problem. The drug that suppresses the illness produces the effect that drives the patient to discontinue it; the patient who discontinues relapses; and each relapse extracts its own toll on function, on the therapeutic relationship, and ultimately on survival [1]. Non-adherence, in other words, is not merely a behavioral footnote to metabolic care - for a substantial share of patients it is downstream of the very weight gain we have treated as a separate issue.

It is into this impasse that glucagon-like peptide-1 (GLP-1) receptor agonists - a drug class that enhances glucose-dependent insulin secretion, suppresses glucagon release, slows gastric emptying, and acts centrally to reduce appetite [3,4] - have arrived, and the evidence in this specific population is no longer preliminary. These agents are conventionally indicated for the management of type 2 diabetes mellitus and for chronic weight management in obesity [3,4]. In clozapine- and olanzapine-treated patients with schizophrenia-spectrum disorder, liraglutide produced clinically meaningful reductions in weight and in dysglycaemia [3]. More recently, the SEMA-PSY trial showed that semaglutide improved metabolic parameters in schizophrenia-spectrum patients with early metabolic abnormalities [4]. In both cases - and the point deserves emphasis - the benefit came without any worsening of psychiatric symptoms [3,4]. For a field long and rightly cautious about introducing new agents into an unstable illness, the accumulating signal of psychiatric safety is as important as the metabolic effect itself.

The conventional reading files these drugs under "metabolic management" [3,4]: a welcome addition to the toolkit, to be deployed once the damage is established. Unlike metformin, which acts primarily on hepatic and peripheral insulin sensitivity, GLP-1 receptor agonists act centrally on satiety regulation and have shown substantially greater efficacy in reversing antipsychotic-induced weight gain in the populations studied to date [3,4]. We propose a different emphasis. Antipsychotic non-adherence is multifactorial, shaped also by impaired insight, cognitive symptoms, sedation, extrapyramidal effects, sexual dysfunction, substance use, socioeconomic barriers, and treatment complexity. Even so, if antipsychotic-induced weight gain is an important and potentially modifiable contributor to non-adherence, then an agent that removes that weight gain is not only a metabolic intervention but an adherence intervention. Reframing these agents this way shifts clinical priority from reactive management of cardiometabolic risk to proactive prevention of treatment discontinuation, with potential downstream reductions in relapse and psychiatric hospitalization [5]. Its most durable contribution may be second-order - not the waist circumference it reduces, but the antipsychotic the patient continues to take because its most resented consequence has been removed. These agents also attenuate the intrusive preoccupation with food, the "food noise," that shapes the subjective burden of treatment [4]. Patient selection matters: the most appropriate candidates are those integrating baseline body mass index, glycaemic status, cardiovascular risk, the trajectory of antipsychotic-associated weight gain, and prior response to conventional measures such as metformin or lifestyle intervention [5], rather than weight gain considered in isolation. Seen this way, the path from GLP-1 receptor agonism to reduced mortality in schizophrenia may run less through any single metabolic number than through the unglamorous fact of sustained treatment [5].

This is a hypothesis, and intellectual honesty requires that it be labelled as one. It applies most directly to patients experiencing substantial weight gain on second-generation antipsychotics or showing early metabolic abnormalities, the population directly studied in the SEMA-PSY trial [4]. Clinically, this intervention is most appropriate for patients exhibiting rapid antipsychotic-induced weight gain, an elevated baseline body mass index, or comorbid metabolic syndrome, particularly those who have shown an inadequate response to first-line strategies like metformin [3,4]. Ideal candidates must also be evaluated for the absence of severe gastrointestinal comorbidities or absolute contraindications, such as a personal history of medullary thyroid carcinoma. No randomized trial has yet made antipsychotic adherence or all-cause treatment discontinuation its primary endpoint, and improvement in antipsychotic persistence remains a hypothesis rather than an established clinical effect of GLP-1 receptor agonist therapy. The argument advanced here rests on mechanism, on convergent trial data [3,4], and on clinical plausibility [5], not on direct demonstration. Mechanistically, reduced antipsychotic-induced weight gain removes one of the effects patients most often cite as a reason to discontinue treatment; convergent trial data from liraglutide and semaglutide studies demonstrate metabolic benefit without psychiatric destabilization [3,4]; and clinical plausibility follows from the broader rationale that side-effect burden is a modifiable driver of treatment persistence in chronic illness [5]. Future trials should incorporate objective adherence measures, time to discontinuation, psychiatric hospitalization, relapse, metabolic outcomes, and patient-reported treatment acceptability as endpoints, to determine whether metabolic improvement truly translates into better psychiatric treatment persistence.

Genuine cautions remain. Contraindications include a personal or family history of medullary thyroid carcinoma and multiple endocrine neoplasia syndrome type 2. Gastrointestinal adverse effects - nausea, vomiting, and diarrhea - are the most common, are usually dose-dependent, and typically attenuate over time [3]. GLP-1 receptor agonists prolong gastric emptying time as part of their core mechanism of action [3,4], and this delayed gastric emptying can alter the absorption of narrow-therapeutic-index agents co-administered with antipsychotics, clozapine chief among them; this pharmacokinetic interaction is inadequately characterized [3]. At present, the concern is largely theoretical for clozapine specifically - clinically meaningful changes in clozapine concentration have not been systematically demonstrated in the available trial data [3] - and therapeutic drug monitoring should be considered during GLP-1 receptor agonist initiation or dose escalation in clozapine-treated patients. Gastrointestinal tolerability, though usually transient, is not trivial in a population already burdened by treatment side-effects, and cost and access threaten to turn these agents into yet another axis of inequity for patients who are already among the most underserved in medicine.

What follows from all this is an agenda rather than a verdict. Prospective psychiatric trials should adopt objective adherence measures, time-to-discontinuation, and long-term relapse rates as primary endpoints, alongside standard metabolic parameters and patient-reported treatment acceptability [5]. Trials in this population should measure the outcomes that actually bear on survival - adherence, discontinuation, and eventually cardiovascular events [1,5] - and should stop treating weight on the scale as a sufficient endpoint. Psychiatry, for its part, should stop outsourcing cardiometabolic risk to other specialties and treat it as core clinical business, with the training and shared-care pathways that it implies. The reflex to regard GLP-1 receptor agonists as weight-loss drugs that happen to be usable in psychiatric patients inverts the true priority. For the patient with schizophrenia, the weight was never the point. Staying alive is the point - and staying on treatment is how they get there.
